# Satisfaction and persistence with vibegron in the first 6 months of overactive bladder treatment: interim results of the phase 4, real-world COMPOSUR study

**DOI:** 10.1186/s12894-025-01742-6

**Published:** 2025-04-02

**Authors:** Roger R. Dmochowski, Eric S. Rovner, Michael J. Kennelly, Diane K. Newman, Keith Xavier, Elizabeth Thomas, Daniel Snyder, Laleh Abedinzadeh

**Affiliations:** 1 Department of Urologic Surgery, Vanderbilt University Medical Center, Nashville, TN USA; 2 Department of Urology, Medical University of South Carolina, Charleston, SC USA; 3 Carolinas Medical Center, Charlotte, NC USA; 4 Perelman School of Medicine, University of Pennsylvania, Philadelphia, PA USA; 5 Urology Partners of North Texas, Arlington, TX USA; 6 Medical Affairs, Sumitomo Pharma America, Inc., 84 Waterford Dr, Marlborough, MA 01752 USA

**Keywords:** Adherence, Adrenergic beta-3 receptor agonist, Anticholinergic, Antimuscarinic, Medication persistence, Micturition, Telemedicine, Urinary bladder, Urinary incontinence

## Abstract

**Background:**

The COMPOSUR study is evaluating vibegron for the treatment of overactive bladder (OAB) in a real-world setting. We report results of a prespecified 6-month interim analysis, assessing patient-reported treatment satisfaction, persistence, safety, and tolerability over the first 6 months after receiving a new prescription for vibegron.

**Methods:**

COMPOSUR (NCT05067478) is a 12-month, phase 4 study of vibegron. Patients were enrolled if they were ≥ 18 years of age with OAB, initiating vibegron after previously receiving anticholinergics (Cohort A) or mirabegron with/without anticholinergics (Cohort B). Satisfaction was assessed via the OAB Satisfaction With Treatment Questionnaire (OAB-SAT-q; domain scores on 0−100 scale, higher scores denoting greater satisfaction). The primary endpoint is the OAB-SAT-q satisfaction domain score. The key secondary endpoints are the percentage of positive responses to OAB-SAT-q questions 1–3 and 11. Additional secondary endpoints include OAB-SAT-q scores for side effects, endorsement, preference, and convenience. Persistence was assessed as an exploratory endpoint. Safety was assessed via adverse events (AEs).

**Results:**

A total of 403 patients were enrolled and initiated treatment with vibegron; 104 patients discontinued the study before month 6, most commonly owing to withdrawal of consent (*n* = 32) and AEs (*n* = 8). Mean (SD) patient age was 56.1 (12.5) years, and 29% were male. Overall, at 6 months, mean (SD) OAB-SAT-q domain scores were 70.0 (21.9) for satisfaction, 84.7 (20.6) for convenience, 91.1 (16.4) for side effects, and 80.4 (20.6) for endorsement. At 6 months, 85.6% (95% CI, 81.1%–90.1%) of patients preferred vibegron to previous OAB treatment. Most patients responded positively to individual OAB-SAT-questions; outcomes were similar from months 1 to 6 and in Cohorts A and B. Persistence with vibegron treatment at 6 months was 73.9% (95% CI, 69.3%–78.0%). Overall, 33.5% of patients experienced a treatment-emergent AE, most commonly (≥ 2% overall) urinary tract infection (4.0%), headache (2.7%), and dizziness (2.2%).

**Conclusions:**

As of the 6-month interim analysis of the COMPOSUR study, most patients receiving vibegron were satisfied with treatment; satisfaction generally persisted from month 1 to 6, regardless of prior OAB treatment received. Treatment with vibegron was generally safe and well tolerated.

**Trial registration:**

ClinicalTrials.gov identifier: NCT05067478; registered: October 5, 2021.

**Supplementary Information:**

The online version contains supplementary material available at 10.1186/s12894-025-01742-6.

## Introduction

Overactive bladder (OAB) is a common urologic condition, characterized by symptoms of urinary urgency, accompanied by frequency and nocturia, with or without urgency urinary incontinence (UUI) [1]. OAB symptoms can be highly bothersome, and patients with bothersome OAB symptoms report reduced work productivity and diminished health-related quality of life (QoL) [2, 3]. Overall, 16.5% of US adults experience symptoms of OAB, including 16.9% of women and 16.0% of men; women with OAB are more likely to experience UUI [4]. Patients with OAB have > 2.5 times greater monthly healthcare costs [5] and increased healthcare utilization (HCRU) [6] compared with similar patients without OAB. OAB symptoms are associated with increased risk of falls and fractures [7], as well as anxiety and depression [8]; these conditions may contribute to increases in HCRU and costs.

American Urological Association (AUA) treatment guidelines include 2 classes of oral pharmacotherapy as treatment options for OAB: anticholinergics and β_3_-adrenergic receptor agonists [1]. Although anticholinergic medications have shown efficacy in treatment of OAB [9], they are associated with bothersome and treatment-limiting side effects including dry mouth, constipation, and blurred vision [9], which may result from systemic inhibition of muscarinic receptors [10]. Most studies have estimated that real-world 6-month persistence with anticholinergic medications is < 50% [11], with > 20% of patients who discontinue their anticholinergic medication indicating that side effects were among the reasons for discontinuation [12]. In addition, multiple recent studies have reported an association between the use of anticholinergic medications to treat OAB and increased risk of incident dementia [13–16]. For this reason, statement 18 of the 2024 AUA guidelines recommends that clinicians should discuss the potential risk for developing dementia and cognitive impairment with the patient when considering an anticholinergic medication for OAB and that a trial of a β_3_-adrenergic receptor agonist is typically preferred before an anticholinergic medication [1]. Additionally, a systematic review of patient preferences and expectations regarding OAB management concluded that patients generally prefer an oral treatment that reduces urgency, frequency, and incontinence episodes; does not affect cognitive function; and is covered by insurance [17].

Vibegron (GEMTESA) is a selective β_3_-adrenergic receptor agonist approved by the US Food and Drug Administration to treat OAB with symptoms of urinary frequency, urgency, and UUI [18]. In the phase 3, randomized, placebo- and active-controlled EMPOWUR trial, treatment with once-daily vibegron 75 mg was associated with significant reductions vs placebo in daily micturitions, urgency episodes, and UUI episodes over 12 weeks of treatment and was safe and well tolerated in patients with OAB [19]. In the phase 3, randomized, placebo-controlled COURAGE trial, once-daily vibegron 75 mg was associated with significant reductions vs placebo in daily micturitions, urgency episodes, nocturia episodes, and UUI episodes over 24 weeks of treatment and was safe and well tolerated among men with OAB symptoms receiving pharmacologic treatment for benign prostatic hyperplasia.

There is limited information on real-world satisfaction with vibegron in patients with OAB who have had prior OAB treatment. Thus, the COMPOSUR study was designed to evaluate vibegron in real-world clinical practice by assessing patient treatment satisfaction, persistence, and safety [20]. We report an interim analysis conducted when collection of 6-month questionnaires was complete and 144 patients had completed the study.

## Methods

### Study population

Participants were initiating a course of vibegron, which was prescribed by the investigator in the course of their routine clinical practice, and had not previously been treated with vibegron. Eligibility criteria followed the US product label for vibegron; participants were adults (≥ 18 years of age) diagnosed with OAB with or without UUI, with symptoms of OAB for ≥ 3 months prior to baseline. In addition, eligible community-dwelling participants had previously been treated for OAB with anticholinergic mediations or mirabegron, lived in the US, and had prescription drug coverage. The study initially enrolled patients with commercial insurance, but the protocol was amended during enrollment to include patients with coverage through either commercial or governmental health insurance, reflecting the inclusion of vibegron in the Medicare formulary. Patients were excluded if they had a history of invasive OAB treatments or urologic surgery in the 6 months preceding the baseline visit, a history of mixed (predominantly stress) urinary incontinence, neurologic conditions associated with OAB symptoms, at risk for urinary retention as determined by the investigator, or had used vibegron before the baseline study visit.

### Study design

The COMPOSUR trial (NCT05067478) is an observational 12-month, real-world, prospective study; details of the design of the COMPOSUR study have been published [20]. All treatments were prescribed and administered at the discretion of the treating clinician according to standard clinical practice; prescriptions were filled at the patients’ pharmacy with commercially available vibegron. Study medication was not provided for free by the sponsor but was obtained through the standard prescription process with associated payments per insurance plan requirements. Patients with commercial insurance were provided with a copay card, which limited their out-of-pocket costs to $25 per fill; however, patients could choose not to use the copay card if their cost with insurance was less. Patients entering the study were assigned to 1 of 2 cohorts: Cohort A included patients who were previously treated with anticholinergic medications for OAB (without any prior exposure to mirabegron); Cohort B included patients who had any history of treatment with mirabegron monotherapy or mirabegron plus solifenacin (with or without any history of using anticholinergic OAB medications other than solifenacin).

Patients were seen in the investigators’ practices or via a virtual visit at baseline (within 7 days prior to the first dose of vibegron); were contacted by phone 2 weeks later to confirm that they had started study medication; and had follow-up visits (in clinic or virtual) at 4–6 weeks, 12–20 weeks, and 24–36 weeks, according to the investigator’s usual practice policy regarding follow-up for patients with OAB. Follow-up visits assessed pregnancy status, concomitant medications, adverse events (AEs), patient-reported treatment persistence, and reasons for discontinuation. Because this was a real-world study with a commercially available drug, pill counts were not performed, and treatment compliance was assessed as reported by patients. At the baseline visit, patients signed up for an online portal, and email reminders with a link to the portal were sent monthly for 12 months for patients to fill out the OAB Satisfaction With Treatment Questionnaire (OAB-SAT-q) and report pregnancy status, new concomitant medications, and treatment compliance, including whether they were still taking vibegron. Monthly patient portal windows were open for patients to input responses for 2 weeks after the reminder email (± 1 week of the nominal time point). Patients who indicated via the portal that they had discontinued vibegron were instructed to contact the site to discontinue the study.

### Assessments

Patient demographics, medical history, and medication information were obtained at the baseline visit. Patients completed the OAB-SAT-q monthly after treatment initiation via the online portal. The OAB-SAT-q comprises 11 questions: questions 1–5 and 11 are scored on a 6-point Likert scale; questions 6–8, on a 5-point Likert scale; and questions 9 and 10, on a 4-point Likert scale. Patients completed the OAB questionnaire short form (OAB-q) at baseline and monthly after treatment initiation. Persistence was assessed as the time from initiation to patient-reported date of vibegron discontinuation or time of censoring (last treatment date on study) for patients who reported that they were still taking study treatment at the 6-month time point. Treatment discontinuation date was collected at the end of study visit. Patients were imputed as discontinued if they did not have a discontinuation date recorded at their last study visit but reported discontinuing vibegron via the portal at a monthly patient contact; patients were imputed as censored at the time of last patient contact if they neither had a discontinuation date recorded at their last study visit nor reported discontinuation via the portal. Patients with no postbaseline data from the electronic portal were censored at day 1. Safety was assessed via clinical review of AEs and reasons for discontinuation, collected at each monthly contact and study visit.

### Endpoints

The primary endpoint was patient satisfaction with vibegron treatment at month 12, assessed via the OAB-SAT-q satisfaction domain. Satisfaction was assessed as a composite of the OAB-SAT-q questions 1–3 and normalized to a score range of 0 (extremely dissatisfied) to 100 (extremely satisfied). Additionally, the percentage of patients consistently satisfied with treatment (ie, selecting satisfied or better on all 3 questions) was assessed.

The key secondary endpoints were the percentage of patients who responded positively (satisfied, very satisfied, or extremely satisfied) to individual OAB-SAT-q questions 1, 2, 3, and 11. Additional secondary endpoints included OAB-SAT-q scores for the convenience, side effects, preference, and endorsement domains; safety assessed via the incidence of AEs; and the number of and reasons for treatment discontinuations. Persistence with vibegron was assessed as an exploratory endpoint. Exploratory endpoints included change from baseline at month 12 in the OAB-q health-related QoL total score and symptom bother score and percentage of patients achieving minimal clinically important difference in each score, the results of which will be presented at the time of the final 12-month analysis.

### Statistical analysis

Prespecified interim analyses occurred when most patients had completed the 3- and 6-month questionnaires. A sample size of 400 enrolled patients (≥ 100 patients per cohort) was estimated to provide for a 95% CI with upper and lower bounds within ± 3.635 of the mean. The study had no formal statistical hypothesis, and no adjustment for multiplicity was performed. Results are presented using descriptive statistics. The full analysis set—all patients receiving ≥ 1 dose of vibegron and with ≥ 1 postbaseline assessment of the satisfaction score—was used for all non-safety assessments. The safety analysis set—all patients receiving ≥ 1 dose of vibegron—was used for safety assessments. Persistence was assessed in the safety set using Kaplan–Meier methodology to account for censored observations. Pointwise CIs for persistence at fixed time points were estimated using a complementary log–log transformation [21].

### Ethical conduct

The trial was conducted in accordance with the Declaration of Helsinki, Council for International Organizations of Medical Sciences International Ethical Guidelines, and the International Council for Harmonisation of Technical Requirements for Pharmaceuticals for Human Use. All participants provided written informed consent. The protocol, amendments, and relevant documents were approved by a central institutional review board (Advarra) before initiation. Some study sites required and secured approval from a local institutional review board before initiation.

## Results

### Patients

**Fig. 1:**
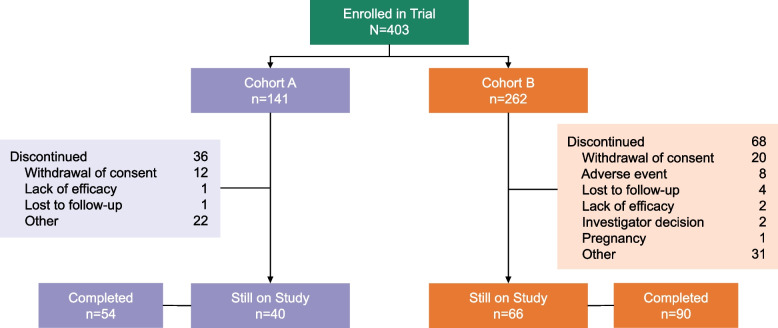
Patient disposition. A total of 403 patients were enrolled. At month 6, 144 patients had completed the trial, and 106 were still in the study. Reasons for discontinuation were collected from 104 patients who discontinued prior to month 6; 49 patients (Cohort A, *n* = 11; Cohort B, *n* = 38) discontinued after month 6. Cohort A = prior anticholinergic; Cohort B = prior mirabegron ± anticholinergic

Patients were enrolled from October 28, 2021, to July 27, 2023. At the time of the 6-month interim analysis, 403 patients were enrolled (Cohort A, *n* = 141; Cohort B, *n* = 262), and enrollment was complete. Overall, 104 patients discontinued the study prior to month 6; reasons for study discontinuation included withdrawal of consent (*n* = 32), AEs (*n* = 8), loss to follow-up (*n* = 5), lack of efficacy (*n* = 3), investigator decision (*n* = 2), and becoming pregnant (*n* = 1) (Fig. 1). Analysis of patient-reported reasons for discontinuation during the study remains ongoing.

Mean (SD) age was 56.1 (12.5) years, and 29% of patients were male, 51.3% of whom had a history of benign prostatic hyperplasia. Demographic and clinical characteristics were generally well balanced between Cohorts A and B (Table 1). Patients in Cohort A (prior anticholinergic only) were more likely than patients in Cohort B (prior mirabegron) to report discontinuing their prior OAB treatment owing to lack of efficacy (71.6% vs 58.0%, respectively) and less likely to report discontinuing prior treatment owing to safety (4.3% vs 7.3%) or cost (7.1% vs 27.5%).

**Table 1:** Demographics and baseline clinical characteristics (safety analysis set)

Characteristic	Cohort A: prior anticholinergic (*n* = 141)	Cohort B: prior mirabegron (*n* = 262)	Overall (*N* = 403)
Mean (SD) age, y	53.9 (13.7)	57.2 (11.6)	56.1 (12.5)
<65, n (%)	119 (84.4)	207 (79.0)	326 (80.9)
≥ 65, n (%)	22 (15.6)	55 (21.0)	77 (19.1)
Sex, n (%)			
Female	108 (76.6)	178 (67.9)	286 (71.0)
Male	33 (23.4)	84 (32.1)	117 (29.0)
History of BPH^a^	13 (39.4)	47 (56.0)	60 (51.3)
Race, n (%)			
American Indian or Alaska Native	0	1 (0.4)	1 (0.2)
Asian	4 (2.8)	3 (1.1)	7 (1.7)
Black or African American	24 (17.0)	33 (12.6)	57 (14.1)
White	109 (77.3)	214 (81.7)	323 (80.1)
Other^b^	2 (1.4)	6 (2.3)	8 (2.0)
Unknown	2 (1.4)	5 (1.9)	7 (1.7)
Reason for discontinuing prior OAB medication^c^			
Lack of efficacy	101 (71.6)	152 (58.0)	253 (62.8)
Cost	10 (7.1)	72 (27.5)	82 (20.3)
Safety	6 (4.3)	19 (7.3)	25 (6.2)
Other	26 (18.4)	23 (8.8)	49 (12.2)

*BPH* benign prostatic hyperplasia, *OAB* overactive bladder

^a^Presented as the percentage of male patients who had a history of BPH

^b^Includes patients who selected ≥ 2 races

^c^Patients could choose ≥ 1 reason for discontinuation. The reason for discontinuation of the most recent prior OAB medication is reported. Percentages were calculated using the number of patients in the safety set as the denominator

### OAB-SAT-q satisfaction domain scores

**Fig. 2:**
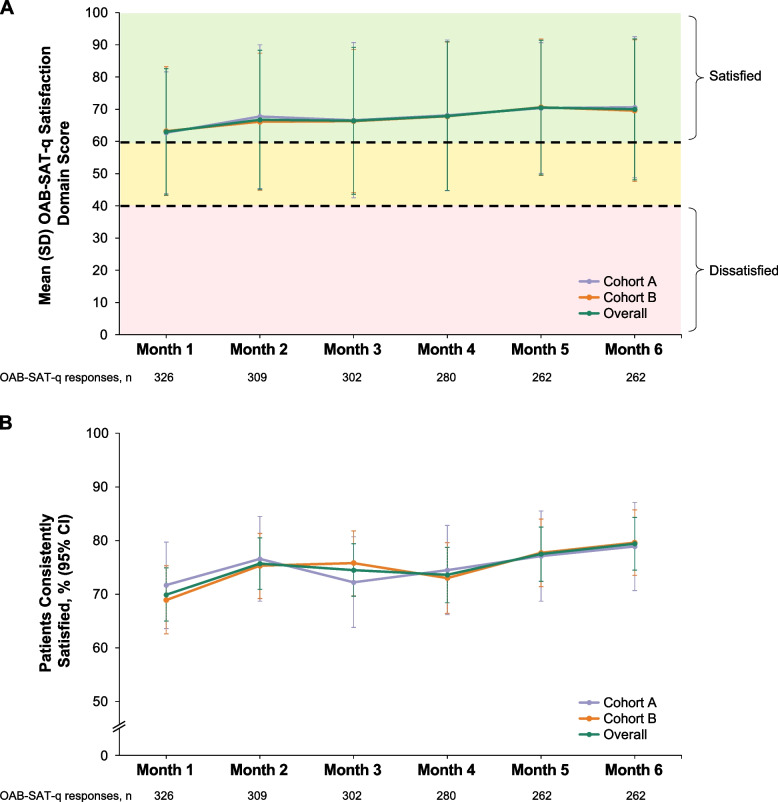
**A** Mean (SD) OAB-SAT-q satisfaction domain score* from 1–6 months after vibegron initiation. **B** Percentage of patients consistently satisfied (responding “satisfied,” “very satisfied,” or “extremely satisfied” to OAB-SAT-q questions 1–3) from 1–6 months after vibegron initiation. *A score of 0 indicates extremely dissatisfied on all 3 questions, and a score of 100 indicates extremely satisfied on all 3 questions. Answering dissatisfied on all 3 questions yields a score of < 40 and answering satisfied on all 3 questions yields a score of > 60. OAB-SAT-q, OAB Satisfaction With Treatment Questionnaire

After 6 months of treatment with vibegron, the overall mean (SD) OAB-SAT-q satisfaction domain score was 70.0 (21.9) (Fig. 2A). Mean (SD) satisfaction domain scores were similar from month 1 to month 6 and among patients in Cohorts A and B at each time point. Overall, 79.4% (95% CI, 74.5%–84.3%) of patients were consistently satisfied with treatment at month 6; the percentages of consistently satisfied patients were similar in Cohorts A and B at each time point (Fig. 2B).

**Fig. 3:**
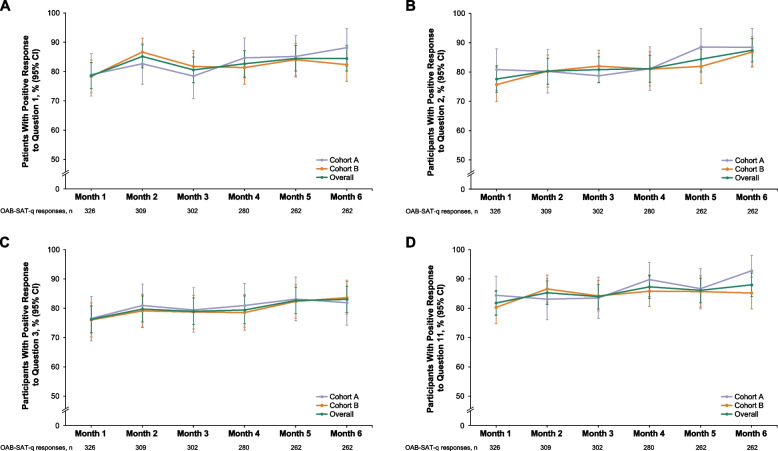
Percentage (95% CI) of patients responding positively to OAB-SAT-q questions (**A**) 1, (**B**) 2, (**C**) 3, and (**D**) 11 at 1–6 months after vibegron initiation. Responses of “satisfied,” “very satisfied,” and “extremely satisfied” were considered positive. OAB-SAT-q, OAB Satisfaction With Treatment Questionnaire

The majority (≥ 75%) of patients in both cohorts responded positively to individual OAB-SAT-questions 1–3 and 11 at each time point; responses were generally similar in Cohorts A and B (Fig. 3; Supplementary Figs. 1–4). Overall, 77.6% of patients responded as at least satisfied with the amount of time it took vibegron to start working after 1 month of vibegron treatment (Fig. 3B; Supplementary Fig. 2).

### OAB-SAT-q convenience, side effects, preference, and endorsement domains

**Fig. 4:**
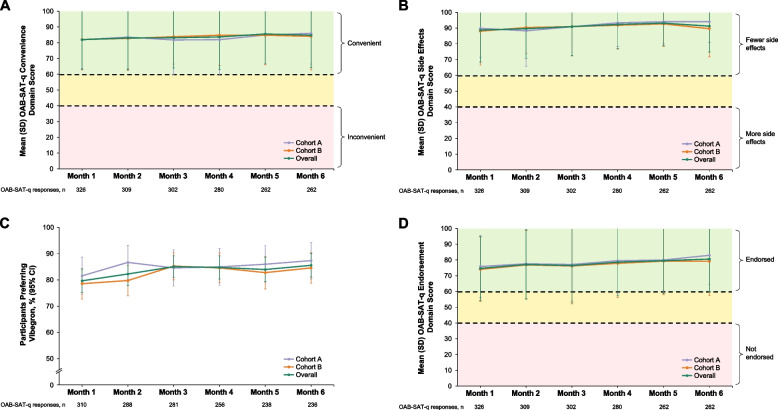
Mean (SD) OAB-SAT-q scores for the (**A**) convenience* and (**B**) side effects* domains from 1–6 months after vibegron initiation. **C** Percentage^†^ (95% CI) of patients preferring vibegron to previous OAB treatment from 1–6 months after vibegron initiation. **D** OAB-SAT-q endorsement* domain score from 1–6 months after vibegron initiation. *OAB-SAT-q domain scores range from 0–100. For the treatment and endorsement domains, higher scores indicate higher levels of treatment endorsement and convenience. For the side effects domain, higher side effect scores indicate fewer side effects. ^†^Patients who selected that they were never before treated for OAB were excluded from the analysis. OAB-SAT-q, OAB Satisfaction With Treatment Questionnaire

After 6 months of treatment with vibegron, the overall mean (SD) OAB-SAT-q score for the convenience domain was 84.3 (20.6); scores were generally similar in Cohorts A and B and at each time point (Fig. 4A). The mean (SD) OAB-SAT-q score for the side effects domain was 91.1 (16.4); scores were generally similar in Cohorts A and B and at each time point (Fig. 4B). After 6 months, 202 of 236 patients (85.6%; 95% CI, 81.1%–90.1%) who filled out the OAB-SAT-q preferred vibegron to their previous OAB treatment; results were generally similar between cohorts and at each time point (Fig. 4C). The overall mean (SD) OAB-SAT-q endorsement domain score was 80.4 (20.6) at month 6; results were similar in each cohort and at each time point (Fig. 4D).

### Persistence with vibegron

**Fig. 5:**
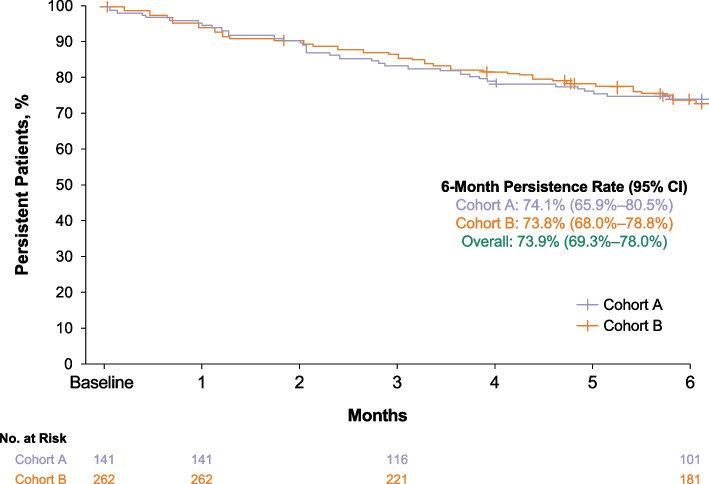
Persistence with vibegron over 6 months following treatment initiation. Patients who did not discontinue treatment were censored at the last known dose date

Overall, 73.9% (95% CI, 69.3%–78.0%) of patients reported that they were persistent with vibegron at 6 months (Fig. 5). Patient-reported persistence rates at 6 months were similar in Cohort A (74.1% [95% CI, 65.9%–80.5%]) and Cohort B (73.8% [68.0%–78.8%]). Patient-reported persistence declined slowly over time; 94.5% (95% CI, 91.7%–96.3%) and 84.9% (95% CI, 81.0%–88.0%) of patients were persistent after 1 month and 3 months, respectively.

### Safety

Overall, 33.5% of patients experienced ≥ 1 treatment-emergent AE (TEAE) (Table 2). The most commonly reported TEAEs (occurring in ≥ 2% of patients overall) were urinary tract infection (4.0%), headache (2.7%), and dizziness (2.2%). Serious AEs occurred in 1.5% of patients overall, and no serious AEs were considered treatment related by the investigator. TEAEs leading to discontinuation of vibegron occurred in 7.2% of patients overall; no deaths occurred as of the data cutoff. AEs of urinary retention occurred in 1.0% of patients, and AEs of hypertension (including essential hypertension) occurred in < 1% of patients overall.

**Table 2:** Summary of safety (safety analysis set)

Patients with TEAE, n (%)	Cohort A: prior anticholinergic (*n* = 141)	Cohort B: prior mirabegron (*n* = 262)	Overall (*N* = 403)
≥ 1 TEAE	53 (37.6)	82 (31.3)	135 (33.5)
≥ 1 treatment-related TEAE	19 (13.5)	42 (16.0)	61 (15.1)
≥ 1 serious TEAE	4 (2.8)	2 (0.8)	6 (1.5)
≥ 1 TEAE leading to treatment discontinuation	10 (7.1)	19 (7.3)	29 (7.2)
TEAEs occurring in ≥ 2% of patients in either cohort^a^			
Urinary tract infection	4 (2.8)	12 (4.6)	16 (4.0)
Headache	2 (1.4)	9 (3.4)	11 (2.7)
Dizziness	1 (0.7)	8 (3.1)	9 (2.2)
Dysuria	3 (2.1)	4 (1.5)	7 (1.7)
Nausea	4 (2.8)	2 (0.8)	6 (1.5)
Upper respiratory tract infection	4 (2.8)	2 (0.8)	6 (1.5)
Hypertonic bladder	4 (2.8)	1 (0.4)	5 (1.2)
Fatigue	3 (2.1)	2 (0.8)	5 (1.2)
Hot flush	3 (2.1)	2 (0.8)	5 (1.2)

*TEAE* treatment-emergent adverse event

^a^Reported adverse events that were not yet coded at the time of the 6-month analysis (*n* = 6) are not presented

## Discussion

COMPOSUR is an observational study, performed in a US practice setting, designed to provide insights into the outcomes of vibegron treatment, as reported by real-world patients with OAB [20]. This 6-month interim analysis of COMPOSUR was prespecified, with the intent of assessing patient-reported satisfaction and persistence with vibegron over the first 6 months following treatment initiation. Notably, treatment satisfaction in COMPOSUR was generally high early in the study and was maintained over 6 months of treatment. The overall mean (SD) satisfaction domain score was 63.0 (19.6) at month 1 and remained ≥ 63 through month 6. At each patient contact from months 1 to 6, > 75% of patients responded positively to each individual OAB-SAT-q question assessing satisfaction. Scores on the OAB-SAT-q convenience, side effects, and endorsement domains were also generally high 1 month after initiating treatment and were maintained through 6 months. Importantly > 75% of patients in both cohorts reported preferring vibegron to their previous OAB treatment at all time points. Notably, OAB-SAT-q scores in each domain were consistent (differing by < 5 points on a 100-point scale at all time points assessed) between patients in Cohort A (who had previously received treatment with anticholinergics) and Cohort B (whose prior treatment included mirabegron).

Real-world studies of treatment satisfaction among patients receiving a β_3_-adrenergic receptor agonist have been reported [22–24]. BELIEVE, an observational study conducted in Europe, measured satisfaction with mirabegron over 12 months using the Treatment Satisfaction–Visual Analog Scale (TS-VAS), which reports satisfaction as a single score on a 10-point scale [22]. Improvements from baseline in TS-VAS scores were reported 2–4 months and 10–12 months after treatment initiation, with a higher percentage of patients showing improvement from baseline on the TS-VAS at 10–12 months than at 2–4 months; notably persistence at the 10- to 12-month time point was 53.8% [22], suggesting that dissatisfied patients leaving the study may have contributed to increased TS-VAS scores. In FAVOR, a 12-week, prospective, open-label study of satisfaction with mirabegron among adult patients with OAB who were dissatisfied with previous anticholinergic treatment, satisfaction was assessed using a single question from the OAB-SAT-q; 63% of patients responded positively after 4 weeks of treatment, and 69% responded positively after 12 weeks of treatment [23]. In a real-world retrospective study of patients with OAB and UUI or mixed urinary incontinence who were prescribed a new course of mirabegron (no patients were reported to have previously received mirabegron) at a single hospital in the UK between May 2013 and April 2014 and contacted in November 2014, treatment satisfaction was analyzed using the OAB-SAT-q, without assessing time since treatment initiation; 39% of respondents were satisfied with mirabegron treatment, and 32% preferred mirabegron to their previous OAB treatments [24]. Importantly, direct comparison of this study to prospective trials is limited by its retrospective design and the potential for recall bias. The results of the COMPOSUR study—with > 75% of participants who completed the OAB-SAT-q responding positively to each question assessing satisfaction after 1 month of treatment and 79.4% of patients being consistently satisfied with treatment at month 6—suggest that early and longer-term satisfaction with vibegron is high relative to satisfaction reported in other real-world studies of β_3_-adrenergic agonists.

Oral pharmacotherapy for OAB has historically been associated with high rates of discontinuation shortly after treatment initiation [11]. A claims analysis of 2496 patients in the California Medicaid program reported that 37% of patients initiating an anticholinergic OAB treatment had no refill in the 6 months after the index prescription [25]. Additional studies are needed to assess persistence with OAB medications in first month after initiation in other patient populations. A survey of patients who discontinued OAB treatment found that approximately two-thirds of patients indicated discontinuing for ≥ 2 reasons, including treatments not working as expected (46%) and experiencing side effects (21%) [12]. Taken together, these results suggest that patient satisfaction or dissatisfaction with multiple aspects of their OAB treatment within the first months following treatment initiation may be an important determinant of treatment persistence and, ultimately, meaningful improvement in OAB symptoms and QoL. Notably, out-of-pocket costs may be an important determinant of real-world medication persistence; in COMPOSUR, 20.3% of patients overall (Cohort A, 7.1%; Cohort B, 27.5%) cited cost as a reason for discontinuing their prior OAB medication.

Overall, 73.9% of patients were persistent with vibegron treatment after 6 months; persistence was similar in Cohorts A and B. The COMPOSUR study assessed persistence as reported by patients at monthly contacts; thus, persistence rates observed using this methodology may not be directly comparable to persistence rates observed in studies based on pharmacy claims. Previous studies assessing patient-reported persistence with β_3_-adrenergic receptor agonists have shown variable results. In PERSPECTIVE, a registry study of patients in the US and Canada with pharmacologically treated OAB, 6-month patient-reported persistence with anticholinergic OAB medications (75.0%) and mirabegron (71.6%) was generally similar to the 6-month persistence observed in COMPOSUR [26]. Importantly, PERSPECTIVE included patients who had previously received mirabegron and anticholinergics [26], whereas all patients in COMPOSUR were receiving vibegron for the first time, precluding direct comparison between the studies. In contrast to PERSPECTIVE, a retrospective study of patients in the UK who were prescribed mirabegron at a single hospital found that patient-reported 6-month persistence with mirabegron was 48%; among those who discontinued mirabegron, 62% reported dissatisfaction secondary to unmet treatment expectations. Notably, a recent retrospective claims analysis of commercially insured patients with OAB in the Optum Research Database found that patients who received vibegron had longer persistence compared with matched cohorts of patients who received mirabegron (median [95% CI], 171 [159–182] vs 128 [122–137] days, respectively; *P* < 0.001) or anticholinergics (172 [159–183] vs 91 [91–91] days; *P* < 0.001) [27].

Vibegron was generally safe and well tolerated over 6 months of real-world use, and no new safety signals were observed. No serious treatment-related AEs occurred. The rate of TEAEs leading to discontinuation of vibegron (7.2%) was low relative to the overall rate of discontinuation, suggesting that patients tolerated the treatment. Thus, other reasons, such as cost, patients lost to follow-up, or administrative causes, were more likely to contribute to the overall discontinuation rate. The most common AEs—urinary tract infection, headache, and dizziness—were among the most common AEs reported in the EMPOWUR trial [19]. Rates of AEs of urinary retention and hypertension were low, occurring in 1.0% and < 1.0%, respectively, of patients overall. Of note, mean age of patients in COMPOSUR was 56.1 years, and data from the 2017–2018 National Health and Nutrition Examination Survey indicate that the prevalence of hypertension is 54.5% among adults 40–59 years of age and 74.5% among adults ≥ 60 years [28]. In addition, retrospective claims analyses have found that hypertension is present at higher rates among patients with OAB than non-OAB controls [29, 30].

This study was subject to several important limitations. Although study participants did not have a history of invasive OAB treatments, they may have received behavioral or conservative treatments that could have affected treatment satisfaction. It is also possible that satisfaction in this population would differ from patients receiving OAB treatment for the first time, and satisfaction with vibegron was not compared to satisfaction with other OAB treatments within the same population. The seemingly high discontinuation rate, which is generally comparable with other studies of OAB medications with a duration ≥ 6 months, may limit the interpretation of satisfaction scores. Satisfaction at each time point was measured among patients who remained in the study and completed the OAB-SAT-q; it is therefore possible for these results to be biased if dissatisfied patients were more likely to discontinue the study or less likely to complete the OAB-SAT-q. Notably, nearly three-quarters of patients remained on treatment after 6 months, and 87.6% of patients (262 of 299) who were still on the study completed the OAB-SAT-q at month 6. Furthermore, changes in OAB-SAT-q domain scores between month 1 and month 6 were modest. Taken together these data suggest that any effect of patients leaving the study between months 1 and 6 was relatively small in magnitude. An intent-to-treat analysis with last observation carried forward will be conducted when final results of the COMPOSUR study, reporting patient satisfaction over 12 months, are available.

## Conclusions

In this 6-month interim analysis of the real-world COMPOSUR study, most patients receiving vibegron were satisfied with treatment by 1 month after treatment initiation, and satisfaction was generally maintained through month 6. Most patients, including those previously treated with anticholinergic medications and mirabegron, preferred vibegron to their previous OAB treatment. Patient-reported persistence was 73.9% after 6 months. Vibegron was generally safe and well tolerated, with no new safety signals identified.

## Supplementary Information

Supplementary Material 1.

## Data Availability

The data that support the findings of this study are available from the corresponding author on a case-by-case basis upon reasonable request.

## Abbreviations

AE: Adverse events
BPH: Benign prostatic hyperplasia
AUA: American Urological Association
HCRU: Healthcare utilization
OAB: Overactive bladder
OAB-SAT-q: OAB Satisfaction With Treatment Questionnaire
QoL: Quality of life
TEAE: Treatment-emergent adverse event
TS-VAS: Treatment Satisfaction–Visual Analog Scale
UUI: Urgency urinary incontinence
