# Supplementary material for: Satisfaction and persistence with vibegron in the first 6 months of overactive bladder treatment: interim results of the phase 4, real-world COMPOSUR study
Source: BMC Urol. 2025 Apr 2;25:66. doi: 10.1186/s12894-025-01742-6 (PMC11963645; doi:10.1186/s12894-025-01742-6)

# Supplementary Appendix

Satisfaction and Persistence With Vibegron in the First 6 Months of Overactive Bladder Treatment: Interim Results of the Phase 4, Real-World COMPOSUR Study

**Authors**: Roger R. Dmochowski, Eric S. Rovner, Michael J. Kennelly, Diane K. Newman, Keith Xavier, Elizabeth Thomas, Daniel Snyder, Laleh Abedinzadeh

Supplementary Figure 1. Percentage of patients selecting each response to Overactive Bladder Satisfaction With Treatment Questionnaire question 1: “How satisfied or dissatisfied are you with the way the treatment relieves your symptoms?”


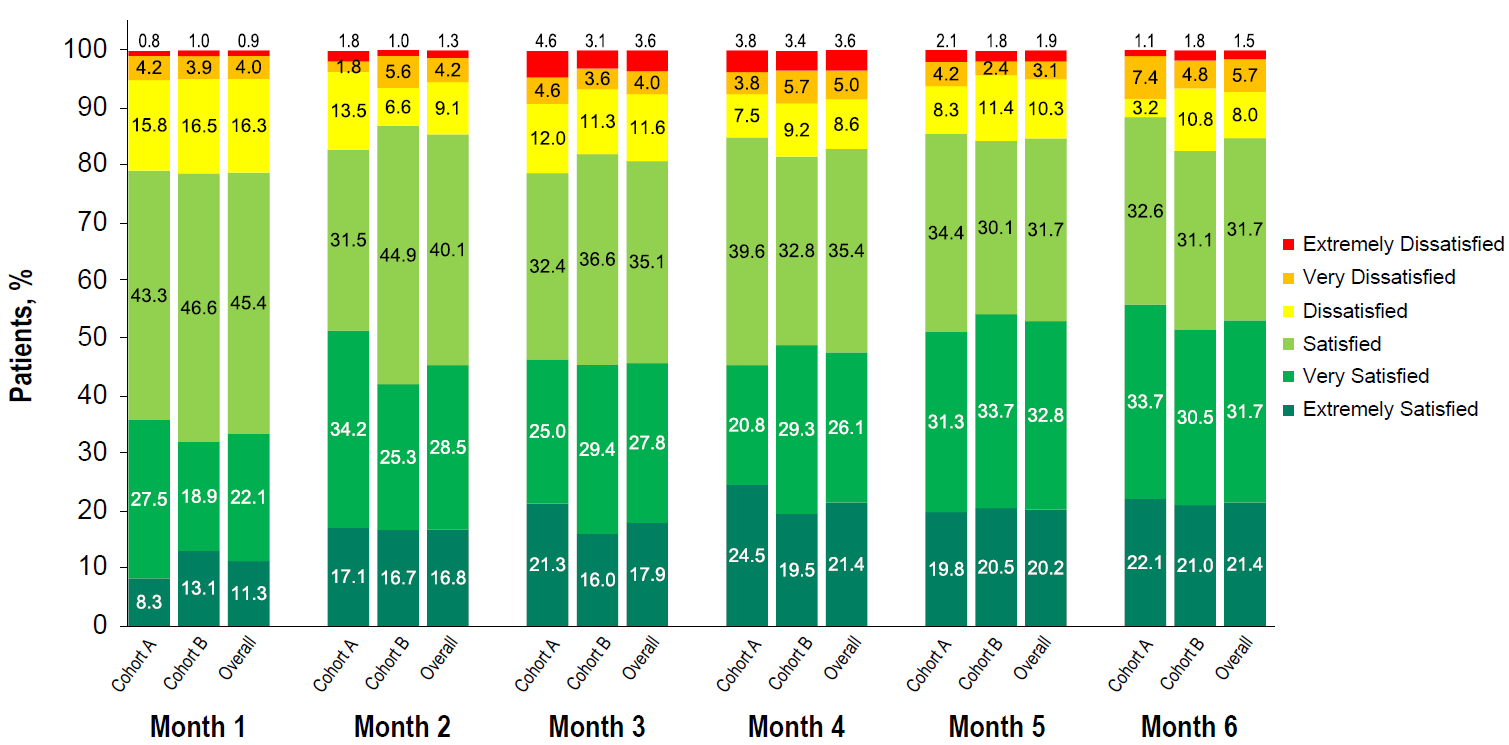


Supplementary Figure 2. Percentage of patients selecting each response to Overactive Bladder Satisfaction With Treatment Questionnaire question 2: “How satisfied or dissatisfied are you with the amount of time it takes the treatment to start working?”


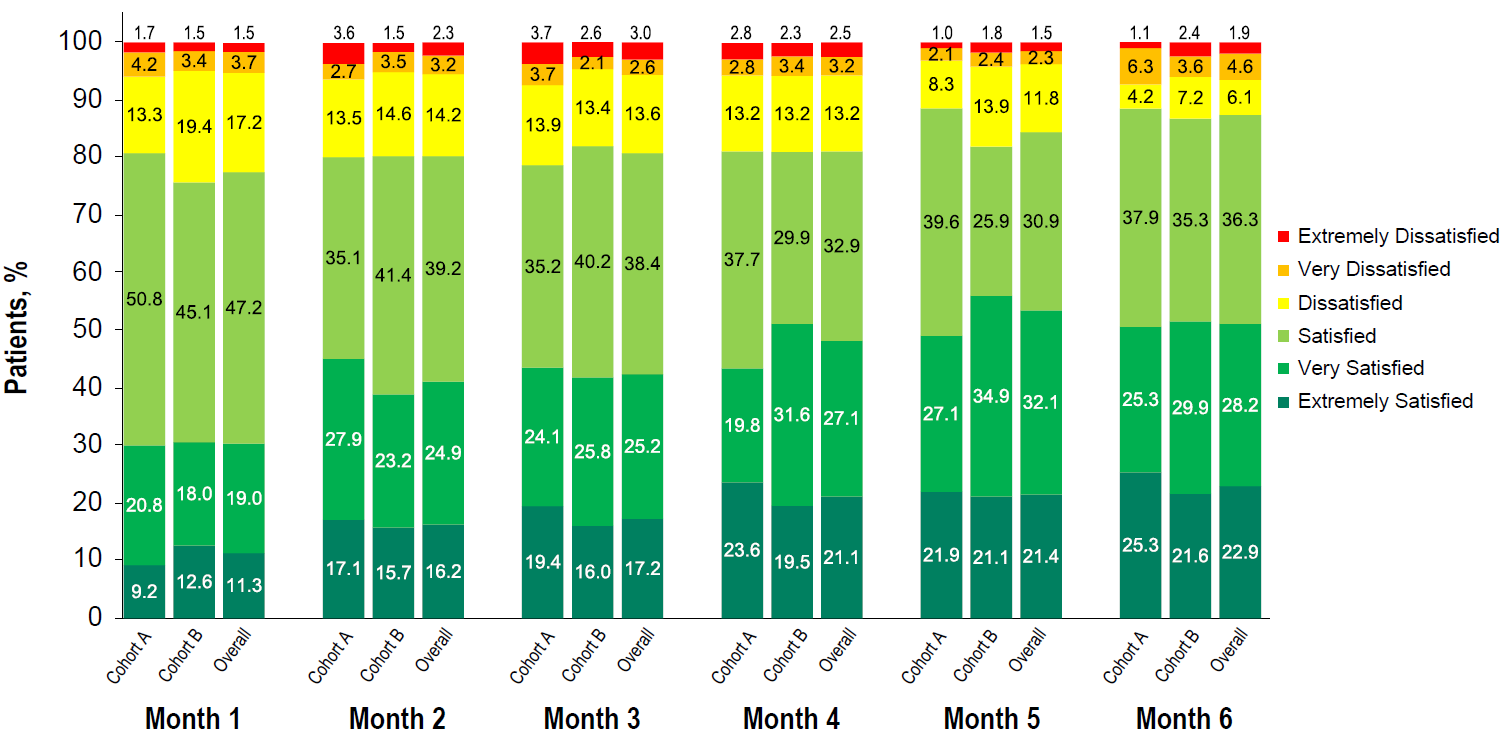


Supplementary Figure 3. Percentage of patients selecting each response to Overactive Bladder Satisfaction With Treatment Questionnaire question 3: “How satisfied or dissatisfied are you with the effectiveness of the treatment, compared to what you expected?”


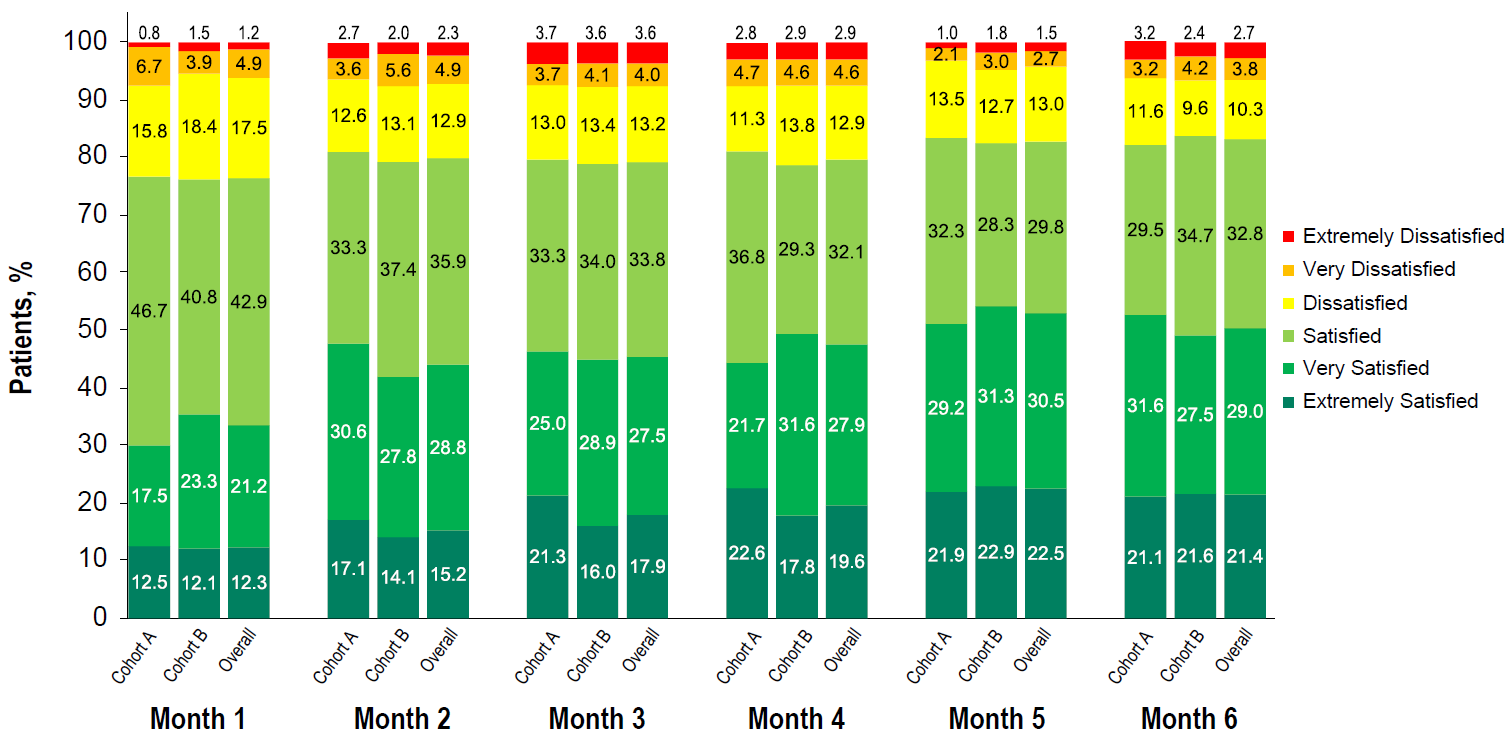


Supplementary Figure 4. Percentage of patients selecting each response to Overactive Bladder Satisfaction With Treatment Questionnaire question 11: “Overall, how satisfied or dissatisfied are you with this treatment?”
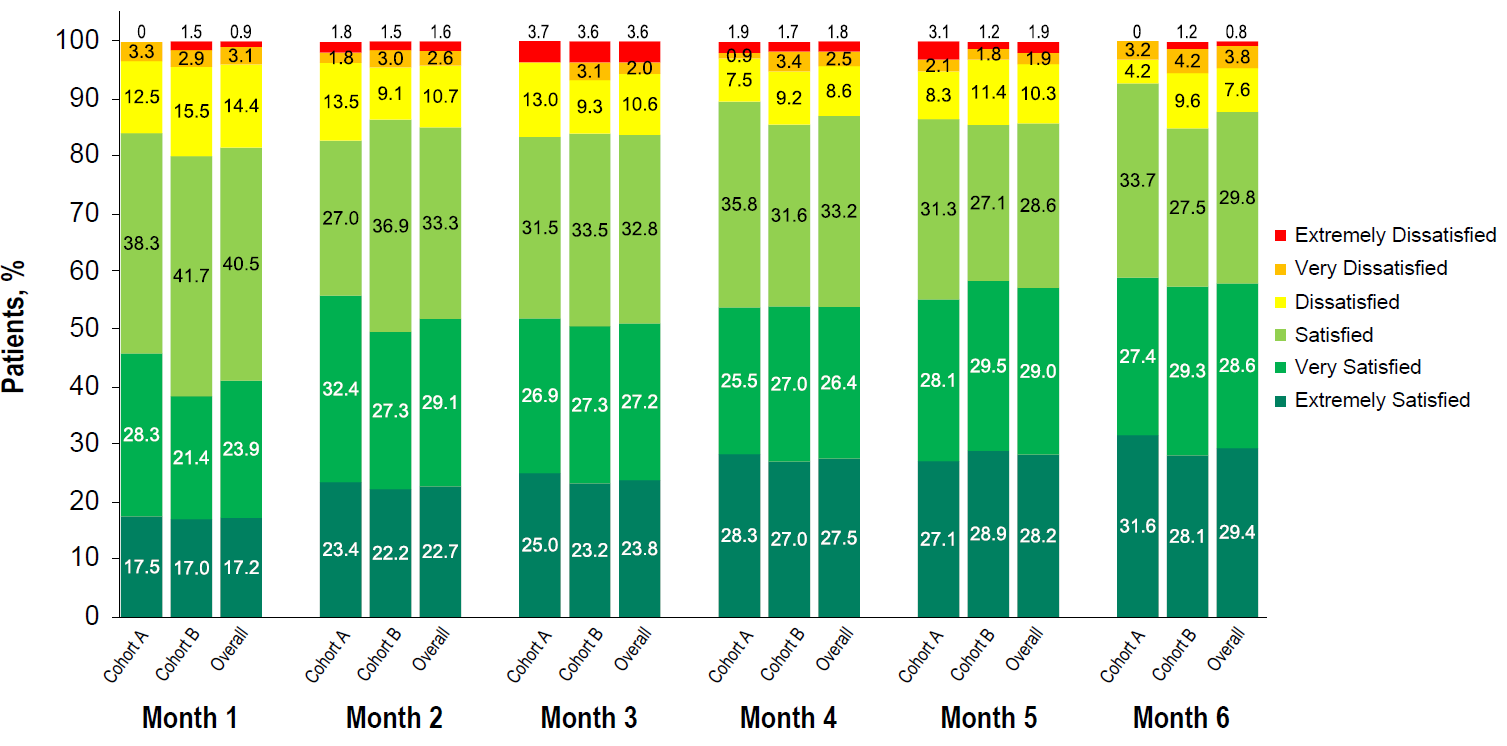

Supplement: Supplementary file 1 — Supplementary Material 1. [file 12894_2025_1742_MOESM1_ESM.docx]
